# Ovarian endometrioma in the adolescent: a plea for early-stage diagnosis and full surgical treatment

**DOI:** 10.1007/s10397-014-0877-x

**Published:** 2015-01-13

**Authors:** Stephan Gordts, Patrick Puttemans, Sylvie Gordts, Ivo Brosens

**Affiliations:** Leuven Institute for Fertility & Embryology, Tiensevest 168, 3000 Leuven, Belgium

**Keywords:** Ovarian endometriosis, Adolescence, Surgery, Transvaginal, Laparoscopy

## Abstract

The incidence and severity of endometriosis in adolescent are comparable with the incidence in adult women. The mean delay between the onset of symptoms and the final diagnosis varies between 6.4 and 11.7 years. The longer the diagnosis is delayed, the more the endometriosis can progress to a more severe stage certainly in the group of patients with pelvic pain. The evolution of endometriosis and its progressivity are not predictable, and the severity of the disease is not directly related to the degree of pain. Endometriotic cysts have a detrimental effect on the ovarian reserve by the evolution in time and the surgical excision technique. Already, in small endometriotic cysts (<4 cm), loss of follicular reserve is present together with the formation of fibrosis in the cortex of the ovary. Early diagnosis of endometriosis in the adolescent deserves our full attention. Non-invasive imaging techniques like 2-D and 3-D ultrasound are helpful in the early diagnosis. Early ablative surgery is recommendable. Although laparoscopy is traditionally recommended, transvaginal laparoscopy has been shown to be most effective in ablating endometriomas with a maximum diameter of 3 cm. Early detection and intervention will contribute to a better quality of life in these adolescents and also to a lower damage of the ovarian tissue by a less invasive ablative surgery.

## Introduction

Ovarian endometriosis, a disease similar to the Mona Lisa face, fails to be grasped and identified by current descriptions. At present, the diagnosis requires an invasive surgical technique, whether laparotomy or laparoscopy, to diagnose the presence of ectopic endometrium-like tissue. In the young woman, the symptoms may be suggestive, but vary greatly and elicit frequent compassion rather than investigation and treatment. Nevertheless, understanding endometriosis in the young woman may shed light on the more complex appearance in the adult woman and improve early-stage management [1]. This review will address the recent literature regarding premenarchal and adolescent endometriosis and discuss in particular the inherent risks of the delay in diagnosis and management of ovarian involvement.

## Premenarchal and adolescent endometriosis

Endometriosis is described as premenarchal and distinguished from adolescent when symptoms and lesions occur during the phase of telarche before the menarche.

### Premenarchal endometriosis

Marsh and Laufer [2] identified, in 2005, endometriosis as a cause of chronic pelvic pain in five premenarchal girls without an obstructive anomaly of the reproductive tract. Breast development in the patients ranged from Tanner I to Tanner III, and non-gynecological etiologies for pelvic pain were excluded. All subjects had laparoscopy with the identification of multiple clear and red lesions consistent with stromal endometriosis. Postoperatively, all of the girls had marked improvement of their pelvic pain based on self-reported pain scales. Two of the subjects had subsequent repeat laparoscopies 6 and 8 years after their initial surgery, which revealed classical endometriosis. More cases of endometriosis occurring before or around the time of menarche have been documented. Gogacz et al. [3] described an 11-year-old patient with a left ovarian endometrioma. Her menarche occurred spontaneously 6 months after surgery. Ebert et al. [4] reported on a 9-year-old premenarchal girl with cyclic pelvic pain since her 8th year of life. Multiple clear, red, and vascularized flame-like peritoneal lesions were observed. The resected lesions showed cytogenic stroma, small glands, and pigment-carrying macrophages.

### Adolescent endometriosis

Originally described more than a century ago, endometriosis is thought of as a disease that affects adult women, but there is increasing awareness of its presence in young women. The disorder represents already in the young woman a vague and perplexing entity that frequently results in chronic pelvic pain, adhesive disease, and infertility. Differences exist between adolescent and adult types of endometriosis, but it is likely that diagnosis and treatment during adolescence decrease disease progression and prevent subsequent reproductive failure. The first study of adolescent endometriosis by Hanton et al. [5] covered the Mayo Clinic experience from 1935 until 1964 and included 68 young patients. The authors tried to determine the frequency, relation to menarche, and outcome. Sixty-three (93 %) out of the 68 patients experienced menarche 5 to 10 years before diagnosis, 9 could not date menarche but were 21 or younger at diagnosis, and 6 had congenital obstruction to menstrual flow. Usually, patients complained of dysmenorrhea or other pelvic pain, but 11 (16 %) had no pelvic complaints. Only three complained chiefly of infertility. Ten patients initially had procedures ablating menstrual function and 58 had conservative operations. One month to 25 years after conservative treatment, 15 patients required subsequent operation, radical in 12 cases. Subsequent fertility was about 50 % in this study. Parker and collaborators [6] recently investigated menstrual pain and other symptoms in teenagers to evaluate how many experience a degree of menstrual disturbance that needs to be further investigated. In a population of 1051 girls aged between 15 and 19 years, the authors concluded that menstrual pain and symptoms are common in teenagers. Girls indicating moderate to severe pain in association with a high number of menstrual symptoms, school absence, and interference with life activities should be effectively managed to minimize menstrual morbidity. Those girls who do not respond to medical management should be considered for further investigation for possible underlying pathology, such as endometriosis. In a study of Laufer et al. [7, 8], the prevalence of endometriosis in adolescents with chronic pelvic pain not responding to medical therapy was 69.6 %.

A recent Chinese study by Yang et al. [9] included 63 patients less than 20 years old with surgically diagnosed endometriosis at the Peking Union Medical College Hospital from 1992 to 2010. Mean age at diagnosis was 18.41 ± 1.84 years with a much earlier disease onset in adolescents with genital tract malformations. Of the 35 patients with follow-up time that ranged from 12 to 98 months, 9 in 15 patients discontinued medical treatment after operation and had a recurrence. Seven in 15 patients who took oral contraceptive pills or progestin only pills had recurrence, but none of the five patients receiving gonadotropin-releasing hormone agonist. Among the 15 cases without postoperative medical therapy, all five cases with lesions at multiple sites had recurrence, while only four of the other ten cases had relapse. The difference was of statistical significance (Fisher’s exact test, *P* = 0.044). The authors concluded that the presence of lesions at multiple sites is a risk factor of recurrence and that GnRHa can effectively prevent the recurrence.

Recent studies of endometriosis in adolescents show clearly that the disease is no longer characterized by subtle superficial lesions, but also by the presence of ovarian adhesions and endometriomas [1]. Comparison of the clinical features of the endometrioma in adolescent women to women of other age groups by Lee et al. [10] showed that adolescent females experienced menarche at a significantly earlier age and that the main symptom was pain (77 %). The proportion of incidental detection (23 %) was low in comparison with women older than 30 years. The authors concluded that, apart from pain, there were no other differences between the age groups. Apparently, adolescent endometriosis is a hidden, debilitating, and progressive disease that deserves greater attention for diagnosis and more appropriate management for the preservation of the integrity of reproductive life.

## Views on the pathogenesis of premenarchal endometriosis

The presence of peritoneal and ovarian endometriosis in premenarchal girls without an obstructive anomaly has supported the concept that endometriosis may result from an etiology other than retrograde menses as proposed by Sampson in 1927 [11]. Batt and Mitwally [12] have argued for recognition of embryonic Mullerian rests as the pathogenesis in cases of early endometriosis not explained by accepted theories. Along with John Huffman, a founder of the subspecialty of pediatric and adolescent gynecology in North America, they proposed that telarche be recognized as a developmental benchmark, after which endometriosis is included in the differential diagnosis of chronic pelvic pain. In recent years, Signorile et al. [13, 14] demonstrated the presence of ectopic endometrium in human female fetuses at different gestational ages. They suggested that endometriosis is caused by dislocation of primitive endometrial tissue outside the uterine cavity during organogenesis. Also, Bouquet de Jolinière et al. [15] described the presence of misplaced endometrial glands and embryonic duct remnants in six of seven fetuses referring to the possible theory of involvement of Müllerian or Wolffian cell rests in the pathogenesis of endometriosis.

Following the current available evidence regarding stem/progenitor cells in the human endometrium, Oliveira et al. [16] suggested the possible involvement of these cells in the etiology of endometriosis. The identification of stem cells in animal and human tissues is, however, very complex, and the putative stem cells are supposed to be found through several assays such as clonogenicity, label-retaining cells, “side population” cells, undifferentiating markers, and cellular differentiation. Bone marrow-derived stem cells transplanted into humans and animals have also been identified in eutopic endometrium and endometriotic implants. The actual scientific knowledge obtained on the existence of somatic stem cells in the murine and human endometrium and the implication and biological pathways of these cells in endometriosis has been recently reviewed by Cervello et al. [17]. Recently, Brosens and Benagiano [18] formulated the hypothesis that perinatal uterine bleeding occurring in some newborns—a phenomenon that is routinely discounted as insignificant—may be a cause of premenarchal and adolescent endometriosis. The hypothesis is based on anatomical and functional observations. First, in the perinatal period, the fetal endometrium shows decidualization, shedding, and bleeding in some 5 % of the newborn girls [19, 20]. Secondly, the anatomical structure of the neonatal uterus favors, in contrast with menstrual bleeding, tubal reflux [21]. Thirdly, in premenarchal endometriosis, the sites of implantations have a similar pelvic pattern as in adolescent and adult endometriosis [2]. Fourthly, Arcellana has documented a case of neonatal endometriosis in 1996 [22]. Finally, neonatal and premenarchal lesions with scanty glandular development seem to reflect a stromal endometriosis that later develops into an adolescent and adult type of endometriosis. For these reasons, there is no reason to postulate a different origin and pathogenesis for premenarchal, adolescent, and adult endometriosis. Sampson’s hypothesis can be modified by assuming the perinatal bleeding as the first uterine bleeding with reflux with the shedding of predominantly endometrial stem/niche cells [23].

## Progression of endometriosis

Progression of the endometriotic lesion is characterized by two stages of morphological activities. In the first phase, the superficial endometriotic implant responds like eutopic endometrium to ovarian steroid hormones resulting in proliferation, secretory changes, and decidualization followed by superficial desquamation and bleeding. In the later phase, the interstitial implant is associated with smooth muscle metaplasia and formation of deep or adenomyotic nodules. In the ovary, progression of endometriosis leads to bleeding and adhesions resulting in endometrioma formation, while interstitial smooth muscle metaplasia and fibrosis affect the cortical zone and decrease the follicular reserve.

### Ovarian endometrioma formation

Superficial endometriosis is characterized by adhesion formation. It is now well accepted that the typical ovarian endometrioma is caused by encapsulation of endometrial tissue between the ovarian cortex and the posterior leaf or the parametrium as originally described by Hughesdon in 1957 [24]. In an initial stage, Gordts et al. described the presence of small adhesions only upon the ovarian surface covering the early formation of an invaginating small endometrioma identified as a brownish vesicle [25].

Careful inspection by ovarioscopy [26, 27] allows identification of vascularization and pigmentation of the pseudocyst lining and to distinguish between the endometrioma with a pearl white or yellowish-pigmented cortex lined by a thin mucosa with prominent neoangiogenesis (red endometrioma) and the endometrioma with a dark, pigmented fibrotic tissue (black endometrioma). Occasionally, the endometrioma is connected to a corpus luteum that has a completely different surface, and early colonization by endometriotic surface epithelium can be observed. One can argue that full excision of the red endometrioma represents, in many cases, excessive surgery and that particularly in young adults, in whom fibrosis is absent, this invariably results in the resection of healthy ovarian cortex.

### Cortical and interstitial changes in the endometriotic cyst

One of the subtle and poorly appreciated changes associated with ectopic endometrium is smooth muscle metaplasia (SMM). According to Anaf et al. [28], deep infiltrating endometriosis (deeper than 5 mm under the peritoneum) in adults often takes the form of a nodular lesion (or “adenomyotic nodule”) consisting of smooth muscles and fibrosis with active glands and scanty stroma. They studied in adults 54 endometriotic lesions originating from four different pelvic locations (peritoneum, ovary, rectovaginal septum, and uterosacral ligaments) using a monoclonal antibody against muscle-specific actin for identifying the presence of smooth muscles and quantifying the smooth muscle content. They found that smooth muscles were frequent components of endometriotic lesions in pelvic locations and concluded that, in adults, the definition of distinct endometriotic entities based on the difference in the tissue composition of the lesions (endometriotic nodules versus adenomyotic nodules) is inconsistent with the very frequent presence of smooth muscle cells in endometriosis irrespective of their localization. In a study of ovarian endometriomas, Fukunaga [20] noted that SMM in ovarian endometriosis is not an uncommon phenomenon and assumed that smooth muscle may originate from either metaplastic endometrial stromal cells in endometriotic foci or metaplastic ovarian stromal cells in the rim of endometriosis.

At present, Sampson [11] and Hughesdon [24] are the only investigators to study systematically histological sections of ovaries with endometrioma in situ. Admittedly, the specimens were obtained in older women and, therefore, represent a later stage of the disease. The pathologist Hughesdon described the main features of disruption and disorganization of the cortical wall and loss of identity of the inner cortex by smooth muscle cell metaplasia occurring in any of its layer in 86 % of the chocolate cysts. As a result, the inner cortex may become quite unrecognizable by stretching and muscular metaplasia, and in addition, there is no cleavage plane. It is therefore likely that the main risk of late diagnosis and treatment of the ovarian endometrioma is the progressive structural disorganization of the inner cortex. In the absence of a cleavage plane, surgical reconstruction of the ovary is becoming critically difficult. The changes are not dependent on the size of the pseudocyst. In a study of cystectomy specimens, Scurry et al. [29] noted that the presence of oocyte in such specimens is influenced, in addition to age, by fibrosis, SMM, and stretching of the cortex making identification frequently unrecognizable. Clement [30] noted that a biopsy is required for histological diagnosis, but the diagnostic value is compounded by tissue that is limited to a small biopsy specimen.

### Loss of follicle reserve

The question arises to which extent the SMM associated with the ovarian endometrioma affects the follicular reserve. In a recent study, Kuroda at al. [31] obtained a small amount of normal ovarian tissue during ovarian cystectomy in 61 women with ovarian endometrioma and 42 patients with non-endometriotic cysts. The density of follicles in the ovarian tissues correlated with the age of the patients in both groups, but in women aged <35 years, the relative density of follicles in healthy ovarian tissues was consistently lower in the endometriotic cyst group compared to the non-endometriotic cyst group. The resection rate of normal ovarian tissue in cystectomy specimen of the endometriosis group was significantly higher than in the non-endometriotic cyst group. The authors concluded that ovarian endometriomas have a detrimental impact on follicle reserve in younger patients, and furthermore, that laparoscopic cystectomy for endometriomas may accelerate the rate of oocytes loss associated with aging. This study is important for demonstrating that the impact of endometrioma on the follicular reserve is determined by both the evolution in time and the surgical excision technique. The data support the recommendation of early-stage ablation of the endometriotic implant and avoid both delay of surgery as well as excision technique.

The morphological data on follicle loss in women with endometrioma has been confirmed by the impact of endometrioma on serum anti-Mullerian hormone (AMH), which is proposed as a marker of ovarian reserve. In a retrospective study comparing serum AMH levels in 1642 infertility patients without endometrioma and 141 patients with endometrioma, Hwu et al. [32] found that both ovarian endometrioma and cystectomy are associated with a significant reduction on ovarian reserve. Moreover, the mean serum AMH level was significantly lower in patients with bilateral endometrioma compared to that of patients with unilateral endometrioma. A lower concentration of AMH in patients with ovarian endometriomas before any surgery compared to normal ovaries is also described by Pacchiaroti et al. [33].

Kitajima et al. [34] demonstrated that in ovaries with endometriomas less than 4 cm in diameter, follicular density is significantly lower than in cortex from contralateral normal ovaries. In their 2014 paper [35], a so-called “burnout” hypothesis has been described with an accelerated follicular recruitment and atresia in early follicles found in ovaries with endometriomas and not in the cortex of contralateral ovaries without endometriomas. Focal inflammation results in the structural alteration of the ovarian cortex, with massive fibrosis and loss of cortex-specific stroma. Focal loss of follicular density may be associated with a “vicious circle of dysregulated folliculogenesis that eventually results in a burnout of the stockpile of dormant follicles.”

Fibrosis of the endometriotic cyst was also frequently observed (9/13) in the study of Schubert et al. [36] while this was not present in case of dermoid cyst and serous cyst where the ovarian cortex only seemed to be stretched by the cyst and not damaged.

## Delay in diagnosis

According to the endometriosis literature, the two main reasons why medical advice is sought are chronic pelvic pain and infertility. However, several studies have shown that the onset of symptoms precedes by several years the diagnosis of endometriosis. In a study comparing demographic, epidemiological, and medical data, Dmowski et al. [37] noted that in the pelvic pain group, there was a negative correlation between the age at first symptom and the stage of endometriosis at the time of first diagnosis. Thus, the longer the diagnosis was delayed, the more the endometriosis was in an advanced stage at the time of diagnostic laparoscopy. The frequency of stage IV endometriosis at the time of initial laparoscopy was significantly higher in the pelvic pain group than in the infertile group (31 vs. 12 %). This suggests a more expedient diagnosis in infertile women as compared with those presenting pelvic pain symptoms.

The study also found that the majority of women with endometriosis and infertility had either mild or no pelvic pain symptoms, suggesting the possibility of asymptomatic endometriosis. According to previous studies, unsuspected endometriosis is found in multiparous women undergoing laparoscopic tubal sterilization with a prevalence ranging between 2 [38] and 3.7 % [39]. Hadfield et al. [40] recruited through endometriosis self-help groups a total of 218 women with surgically confirmed endometriosis. US women had a mean ± SD delay in diagnosis of 11.73 ± 9.05 years, while in UK women, the delay of 7.96 ± 7.92 years was significantly lower. Interestingly, American women reported their symptoms to commence some 5 years earlier than British women. Husby et al. [41] reported that the mean delay from the onset of symptoms to diagnosis included two phases of delay: first, a delay from the onset to a doctor visit of 1.4 ± 2.9 years, and secondly, a delay from the doctor visit to establishing a surgical diagnosis of 5.2 ± 5.6 years. It is noteworthy that 21 (6.4 %) of 328 patients with proven endometriosis were diagnosed without pain symptoms. When excluding this group, the mean diagnostic delay would be 6.5 ± 6.3 years and the median delay 5.0 years. Factors influencing the delay in diagnosis included the IVF that can be performed without previously performing laparoscopy and that endometriosis can be present without pain. According to the study of Arruda et al. [42], the interval was dependent on the primary symptom since women with infertility took 4 years to be diagnosed with endometriosis, whereas 7.4 years elapsed from symptoms to diagnosis in patients with pelvic pain. A recent multicenter study performed principally in primary care found a delay of 6.7 years between the onset of symptoms and a surgical diagnosis of endometriosis [43]. The delay was longer in centers where women received predominantly state-funded health care (8.3 vs. 5.5 years) and positively associated with the number of pelvic symptoms (chronic pelvic pain, dysmenorrhea, and dyspareunia) and heavy periods and a higher body mass index. However, it has been documented that menstrual symptoms, while raising a high degree of suspicion for endometriosis, are not reliable as indicators of the severity of disease [44, 45].

## Early-stage management

Early-stage management of endometriosis in the adolescent involves exclusion of reproductive tract anomaly, monitoring the response of pelvic pain to medical treatment and the early ultrasound diagnosis of an endometrioma and, in such cases, full ablative surgery of the ectopic endometrial tissue. The combined oral contraceptive pill has been used for the treatment of endometriosis-associated pain, such as dysmenorrhea for several years. The treatment may also relieve deep dyspareunia, non-cyclic pelvic pain, and dyschezia. While adolescent endometriosis is a hidden, progressive, and severe disease, the medical and surgical tools for diagnosis and treatment should be effective, but minimally invasive.

### Psychological benefits of early-stage diagnosis

Two studies have evaluated in detail the benefits of early diagnosis for the patient. Ballard et al. [46, 47] investigated the reasons why women experience delays in the diagnosis of endometriosis and the impact of this in a qualitative interview-based study of 32 women, 28 of whom were subsequently diagnosed with endometriosis. Delays in the diagnosis of endometriosis occur at an individual patient level and a medical level, as both women and family doctors normalize symptoms, symptoms are suppressed through hormones, and nondiscriminatory investigations are relied upon. Women benefited from a diagnosis because it provided a language in which to discuss their condition, offered possible management strategies to control symptoms, and provided reassurance that symptoms were not due to cancer. Diagnosis also sanctioned women’s access to social support and legitimized absences from social and work obligations. They concluded that although recent guidelines for the management of chronic pelvic pain suggest that diagnostic laparoscopy may be considered a secondary investigation after the failure of therapeutic interventions, the present study highlights the importance of an early diagnosis for women who suffer at physical, emotional, and social levels when they remain undiagnosed.

Nnoaham et al. [43] assessed the impact of endometriosis on health-related quality of life (HRQoL) and work productivity in a multicenter cross-sectional study with prospective recruitment from 16 clinical centers in ten countries. Delay was positively associated with the number of pelvic symptoms (chronic pelvic pain, dysmenorrhea, dyspareunia, and heavy periods) and a higher body mass index. They concluded that endometriosis impairs HRQoL and work productivity across countries and ethnicities, yet women continue to experience diagnostic delays in primary care. A higher index of suspicion is needed to expedite specialist assessment of symptomatic women. Future research should seek to clarify pain mechanisms in relation to endometriosis severity.

It is clear that supportive and comprehensive treatment should be provided until the completion of childbearing.

### Imaging diagnosis of ovarian endometrioma

Transvaginal ultrasound is the first choice for monitoring the ovaries for early-stage development of a uni- or bilateral endometrioma. In the study of Holland et al. [48], the sensitivity and specificity of preoperative ultrasound for the detection of ovarian endometrioma are, respectively, 84.0 (95 % CI 73.7–91.4) and 95.6 (95 % CI 92.8–97.6), although the diameter of the endometriotic cyst was not mentioned. In his paper, Raine-Fenning [49] reported that the results of the predictive value of 2-D ultrasonographic patterns for the detection of endometrioma were very discrepant with a variation of sensitivity and sensibility, respectively, ranging from 64–89 to 89–100 mostly due to inappropriate ultrasonographic diagnosis. In experienced hands, the technique allows reliably the diagnosis of an endometrioma with a size of more than 1–2 cm in diameter. With the use of B-Mode ultrasound and mean gray value, Alcazar et al. [50] reported a sensitivity of 80 (58–92) (95 % CI) and a specificity of 91 (77–97) (95 % CI) with a LR+ of 9.1 (3.0–27.3) and a LR− of 0.2 (0.1–0.5) (95 % CI). In most of the studies reporting the sensitivity and specificity of ultrasound in the differential diagnosis of the pathologic cysts, the mean diameter of the cyst is seldom mentioned, neither the relation of the diameter of the cyst and the accuracy of differential diagnosis. The lowest reported diameter varies between 18 and 24 mm [51–53]. In our consecutive series of 169 patients where endometriosis was diagnosed at transvaginal hydrolaparoscopy (THL), routine preoperative transvaginal ultrasound only detected 45 % of endometriomas smaller than 15 mm [25].

### The place of transvaginal endoscopic surgery

**Fig. 1:**
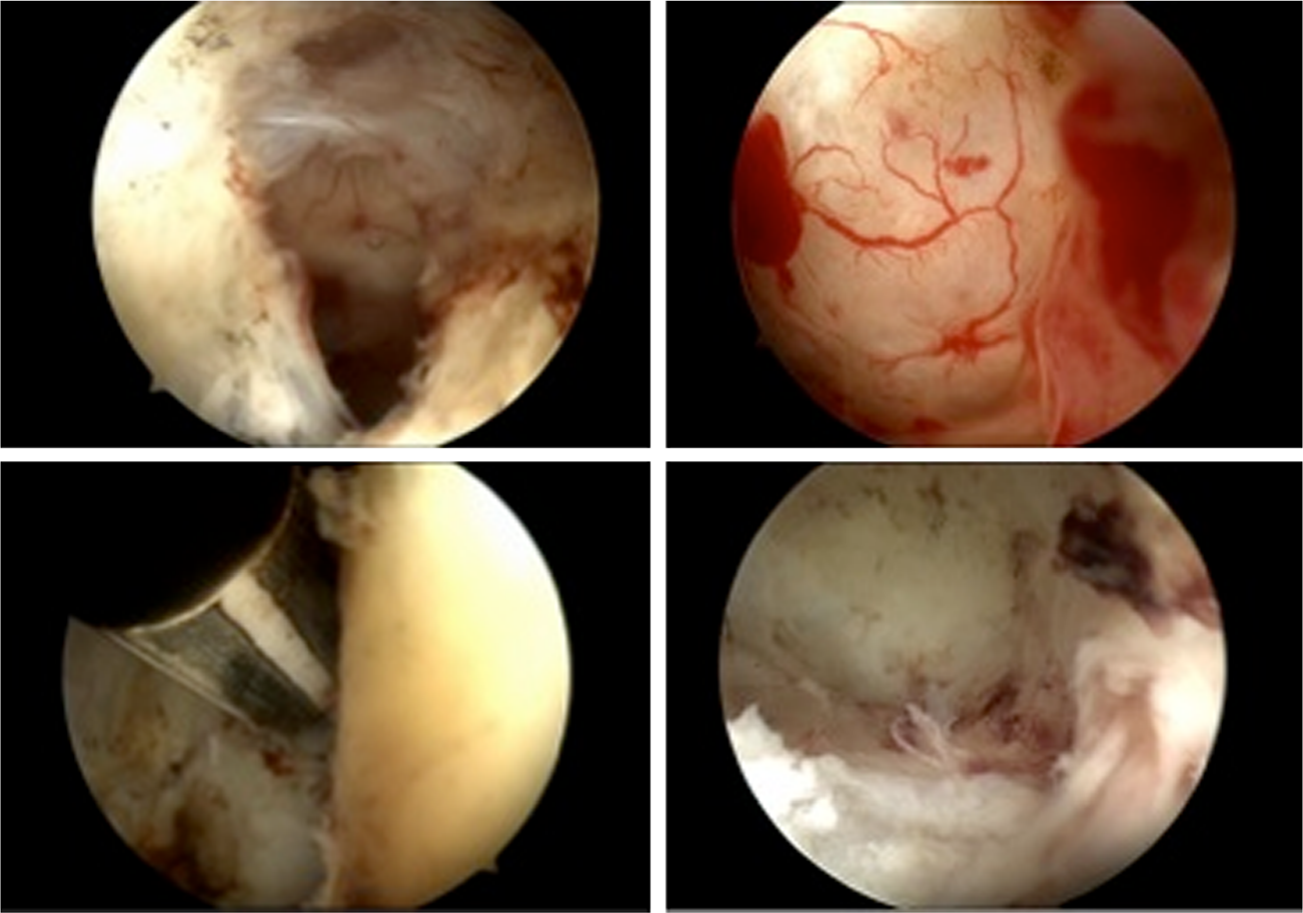
Ablative surgery of small ovarian endometrioma by transvaginal hydrolaparoscopy. From *upper left* to *right under*. Opening of cyst with visualization of microvascularization at the base. Close-up (under water) of insight cyst: remark the pertinent vascularization and the presence of endometrial tissue on the right. Use of bipolar probe for ablative surgery. Final result after ablation: remark the absence of carbonization and the *white color* of the insight comparable with the ovarian cortex

Atraumatic ovarian surgery, to avoid loss of ovarian reserve, is based on early-stage diagnosis when cystectomy can be avoided. Although laparoscopy is traditionally recommended, transvaginal endoscopy has been shown to be safe and most effective in ablating ovarian endometriomas that are not larger than 3 cm in diameter [54, 55]. As the transvaginal laparoscopy is performed using a watery distension medium, it enables accurate visualization of the vascularization of superficial implants and adhesions covering the site of small endometriotic lesions. Apart from the watery distension medium, the supplementary advantage of the transvaginal approach is that it allows inspection of the tubo-ovarian organs in their natural position with easy exploration of the fossa ovarica without the need of manipulating instruments. Brownish vesicles present upon the ovarian surface are by closer inspection small invaginated hemorrhagic lesions in the ovarian cortex covered by thin adhesions. Adhesions can be ablated without extra manipulation. The site of invagination can clearly be identified. By the use of a bipolar needle, the pseudocystic invagination is opened. At the basis of these small invaginations, endometrial-like tissue is identified. After rinsing and identification of the endometrial-like tissue lining the wall, full ablation is easily performed using a 5-Fr. bipolar coagulation probe (Fig. 1). As the whole procedure is performed under hydroflotation, no carbonization occurs and the risk of surgical trauma and adhesion formation is minimal. By close inspection and in the presence of adhesions with the posterior leaf, areas of endometrium-like tissue in the lateral wall can be identified and coagulation is performed. The absence of an elevated intra-abdominal pressure due to the CO_2_ pneumoperitoneum at standard laparoscopy enables not only a better visualization of superficial adhesions and vascularization, but the procedure is also performed in the absence of a status of intra-abdominal hypoxia present at standard laparoscopy. It is questionable if such a long time exposure to the hypoxia caused by the CO_2_ pneumoperitoneum is finally not detrimental for the ovarian reserve and is a co-factor for the diminished AMH concentration after surgery.

**Fig. 2:**
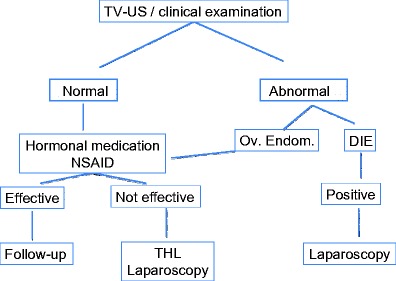
Decision tree in young patients with severe dysmenorrhea. The necessary exploration must be done before referring patient for an operative laparoscopic procedure

In young patients with severe dysmenorrhea, we suggest the following decision tree: In a first step, presence of rectovaginal endometriosis and/or ovarian endometrioma should be excluded by a clinical examination and transvaginal ultrasound. In case of negative examination, patient can be put upon oral contraception and/or NSAID with yearly follow-up; in case of persistence or aggravation of pain, a transvaginal hydrolaparoscopy (THL) or in case of contraindication for a vaginal access standard laparoscopy (SL) should be performed and endometriotic lesions should be treated. In case of diagnosis of ovarian endometriosis in the absence of rectovaginal pathology at clinical examination, a six monthly follow-up under a contraceptive pill is advocated to exclude the increase of the ovarian endometrioma and to evaluate the regression of pain. In case of non-regression of the pain and/or increase of the size of the endometrioma, endoscopic exploration and treatment by THL or SL are mandatory. In the presence of rectovaginal endometriosis, the necessary exploration must be done before referring patient for an operative laparoscopic procedure (Fig. 2).

In the absence of contraindications for a vaginal access [55] and if the size of the endometrioma does not exceed 3 cm, a THL in our hands is preferable. Deep or adenomyotic lesions are rare in adolescents, and as long as there is no evidence, which peritoneal lesions will develop into adenomyotic or deep lesions, there is in our view no indication for “preventive” standard laparoscopy. At THL, the anterior cul de sac cannot be visualized. In asymptomatic patients, however, isolated lesions of the bladder are infrequent [56]. In patients with chronic pelvic pain and DIE, the incidence of bladder endometriosis is reported to be 1.58 % [57] until 6.6 % [58] and patients were complaining of frequent urination and dysuria. It is worth asking whether inspection of the anterior pelvis is necessary in infertility in the absence of tubo-ovarian pathology and in the absence of chronic pelvic pain or urinary complaints. In the presence of clinical symptoms suspicious for bladder endometriosis, appropriate investigation including MRI and standard laparoscopy is advocated. In Finland, the annual incidence, as evaluated from The Finnish Care Register HILMO, increased from 3.6 to 9.4 cases/1,000,000 females aged 15–49 years per year during 1996–1999 and 2004–2007, respectively [59].

### Can endometriosis in the adolescent be cured by full ablation?

At present, there is no evidence that in the adolescent all subtle peritoneal endometriosis can be visualized at laparoscopy. A variety of subtle lesions have been described [60], but the main visual criterion is the presence of microvascularization and cyclic bleeding. For this reason, GnRH agonist can cause disappearance of lesions, but they recur once the treatment is stopped and menstruations follow [61]. A major problem is that, at laparoscopy, the pneumoperitoneum causes collapse of the capillary blood flow inside and surrounding the implants, masking to a large extent the microvascularization in and towards the active subtle lesion. The recent study by Yeung et al. [62] suggested that complete laparoscopic excision of all areas of abnormal peritoneum with typical and atypical endometriosis has the potential to eradicate disease. This publication, however, was criticized by Laufer and Missmer [63] for the statement that the lack of visible endometriosis at second look laparoscopy in a relatively small number of patients was proof of the eradication of disease. As far as peritoneal endometriosis is concerned, the appearance may be variable and hormonal treatment may mask implants. Scanning electron microscopy can show the presence of invisible lesions in women with infertility and endometriosis [64]. Therefore, in the absence of the evidence that ablations of peritoneal endometriosis can cure the disease, the main issue is the full preservation of the ovarian function during reproductive life.

## Discussion

From the early onset of female neonatal life, due to neonatal bleeding and retrograde menstruation, endometrial cells invade the pelvis and are possibly at the origin of the presence of premenarchal and adolescent endometriosis.

It is hard to understand why the diagnosis of endometriosis is so long delayed resulting in severe stages of endometriosis including a frozen pelvis. As health practitioners, we have to question ourselves why we are missing the development of this disease in individual women. Not only the patient herself is unaware of objective symptoms like severe dysmenorrhea and superfluous or frequent menstruation resulting in a late medical advice but also the general practitioners or gynecologist is not sufficiently sensitized for the importance of the symptoms. Furthermore, diagnosis is impaired as there is not a good correlation between the severity of the disease and the symptoms. All these results are a delay of an accurate diagnosis between the onset of symptoms and the final diagnosis. Certainly, in adolescents with chronic pelvic pain such as dysmenorrhea resistant to medication, the incidence of endometriosis is around 70 % [7]. Early ultrasound diagnosis and meticulous follow-up are mandatory. Whether full ablative surgery should be envisaged in an early stage of the disease causing a minimal damage to the ovarian reserve is still debatable and requires further investigation. Long-standing presence of ovarian endometriotic cysts results in SMM and fibrosis of the ovarian cortex, impairing the ovarian reserve. Decisions to operate should be carefully balanced against the growing concern of potential damage of surgery upon the ovarian reserve [65, 66]. It is however questionable if the size of the ovarian endometrioma is of any importance in this decision process. The suggested diameter of 3 cm in the ESHRE guidelines is pure arbitrarily and not based upon any scientific evidence [67]. Alborzi et al. [65] demonstrated that the size of the cyst is inversely proportional with the ovarian reserve. The detrimental effect of the ovarian endometrioma on the follicle concentration and fibrosis, as described in the study of Kitajima et al. [34], was in ovarian endometrioma with a diameter smaller than 4 cm.

The purpose of an early intervention is the treatment of pain, prevention of the progression, and protection of the fertility [68]. As endometriosis is assumed to be a progressive disease, the ACOG recommends the early diagnosis and treatment in the adolescent [69]. In the editorial of Evers [70] evaluating the natural course of the disease in adults, the disease progressed in 29 %, while in the rest of the patients, the disease regressed or stayed stable. The issue of no progression and even disappearance involved mostly peritoneal endometriosis stages I and II, but not ovarian endometriosis [71]. In a prospective cohort study, Alcazar et al. [72] reported a spontaneous disappearance of endometriotic cysts in 30 % of the endometrioma diagnosed by ultrasound. In his study group, however, the mean age of the patients was 40.2 ± 8.5 years and 33.7 % of the patients became menopausal during the follow-up period [mean follow-up time 45 months (9–109 months)]. It remains questionable first if these findings can be extrapolated to the adolescents, where the disease is more aggressive and, secondly, if no visualization at ultrasound means complete resolution of the disease as SMM and fibrosis can go on. In adolescents, the diagnosis is frequently postponed until several years after the onset of symptoms [37, 41, 42]. It is true that, at this moment, the progressivity of the disease cannot be predicted, but the frequent observation of severe stages of endometriosis, even in the absence of pain, is an indication of the progressivity of the disease in the adolescents and deserves our full attention. In a retrospective analysis of postoperative evolution of endometriosis in adolescents, Yang et al. [9] described a recurrence in 53 % of the adolescents, only in the small group of five patients receiving GnRha postoperatively in which no recurrence was observed. A more beneficial effect of acupuncture and Chinese medication in the prevention of recurrence of endometriosis is mentioned in the study of Zhang et al. [73] compared to the use of gestrinone. A higher recurrence rate of endometriosis of 56 % in young women has been mentioned by Tandoi et al. [74], illustrating the degree of aggressiveness of the disease in adolescents.

As ablative surgery of small endometrioma intends to be more accurate and complete, reasonably, we could expect, although not proven, that this will result in a lower recurrence rate, being easier to perform and minimal invasive. Pados et al. [75] and Donnez et al. [76] advice to perform surgery for ovarian endometrioma larger than 5 cm in a two-step procedure enabling more accurate surgery on a smaller cyst in the second time and minimizing the risk of damaging the ovarian reserve.

The use of the transvaginal endoscopic approach adds to the minimally invasiveness of the procedure. The use of a watery distension medium allows an accurate visualization and early detection. Ablative surgery is done using a micro 5 fr bipolar probe causing minimal damage. In the absence of a panoramic view, the transvaginal endoscopic procedure is limited to the treatment of small endometrioma (<3 cm).

Endometriotic cysts differ from other benign cysts as they are extra-ovarian pseudocysts with the absence of a clear delineated capsule and not restricting the disease to the cyst itself but affecting the surrounding cortex by SMM and fibrosis. This explains the difference in ovarian reserve between patients with a dermoid cyst and an endometriotic cyst, with a negative impact on the latter.

## Conclusion

Early diagnosis of endometriosis in the adolescent deserves our full attention. Early ablative surgery can contribute to a lower morbidity, a relief of symptoms, and a better quality of life. Treatment in early stage will result in less damage to the ovary caused by the disease itself and by a less invasive surgical procedure. Endometrial cells on the surface of the ovary carry the risk to affect the ovary in two ways: first by causing ovarian adhesions and pseudocysts and secondly by causing mesenchymal cell metaplasia in the interstitial ovarian tissue, sclerosis, and follicle loss. Similar as in oncology, there is no reason to wait.

Further research has to be done to elucidate if such early treatment will result in lower recurrence rates and less severe forms of the disease.
